# *Aedes aegypti* Mos20 Cells Internalizes Cry Toxins by Endocytosis, and Actin Has a Role in the Defense against Cry11Aa Toxin

**DOI:** 10.3390/toxins6020464

**Published:** 2014-01-28

**Authors:** Adriana Vega-Cabrera, Angeles Cancino-Rodezno, Helena Porta, Liliana Pardo-Lopez

**Affiliations:** 1 Instituto de Biotecnología, Universidad Nacional Autónoma de México, Apdo, Postal 510-3, Cuernavaca 62250, Morelos, Mexico; E-Mails: advcab@ibt.unam.mx (A.V.-C.); helena@ibt.unam.mx (H.P.); 2 Facultad de Ciencias, Universidad Nacional Autónoma de México; Av. Universidad 3000, Coyoacán, Distrito Federal 04510, Mexico; E-Mail: angelescancino@gmail.com

**Keywords:** Cry toxins, endocytosis, actin, insect cells

## Abstract

*Bacillus thuringiensis* (Bt) Cry toxins are used to control *Aedes aegypti*, an important vector of dengue fever and yellow fever. Bt Cry toxin forms pores in the gut cells, provoking larvae death by osmotic shock. Little is known, however, about the endocytic and/or degradative cell processes that may counteract the toxin action at low doses. The purpose of this work is to describe the mechanisms of internalization and detoxification of Cry toxins, at low doses, into Mos20 cells from *A. aegypti*, following endocytotic and cytoskeletal markers or specific chemical inhibitors. Here, we show that both clathrin-dependent and clathrin-independent endocytosis are involved in the internalization into Mos20 cells of Cry11Aa, a toxin specific for Dipteran, and Cry1Ab, a toxin specific for Lepidoptera. Cry11Aa and Cry1Ab are not directed to secretory lysosomes. Instead, Mos20 cells use the Rab5 and Rab11 pathways as a common mechanism, most probably for the expulsion of Cry11Aa and Cry1Ab toxins. In conclusion, we propose that endocytosis is a mechanism induced by Cry toxins independently of specificity, probably as part of a basal immune response. We found, however, that actin is necessary for defense-specific response to Cry11Aa, because *actin*-silenced Mos20 cells become more sensitive to the toxic action of Cry11A toxin. Cry toxin internalization analysis in insect cell lines may contribute to a better understanding to Cry resistance in mosquitoes.

## 1. Introduction

*Aedes aegypti* is the most important vector for the transmission of dengue fever, yellow fever and other tropical diseases. *A. aegypti* is distributed worldwide, and its presence has been increasing in many regions over the past 25 years [1]. One of the most effective control strategies is the elimination of larvae using chemical insecticides [1]. Damage to the environment, human health and other animal species and the emergence of insects that are resistant to chemical insecticides have led to a search for safer alternatives and compounds with higher specificity against mosquitoes.

One of these control strategies involves the use of *Bacillus thuringiensis* Cry toxins. These toxins belong to the pore-forming toxin family (PFT), which constitute the most widespread group of toxins produced by bacteria. These toxins are soluble proteins that exert their functions by binding to specific receptors localized in the membrane of cells of susceptible organisms. After binding, PFT, at high toxin doses, induces death by osmotic shock. However, at low doses, the toxin triggers defense mechanisms that allow cell survival [2]. The defense cell mechanisms triggered by small doses of PFT are less known.

The endocytosis of macromolecules requires the recruitment of various proteins from the cytosol to the plasma membrane, leading to invagination and subsequent excision of the membrane, which forms a vacuole inside the cell. Several pathways involved in endocytosis have already been described, including clathrin-mediated endocytosis (CME), caveolae, phagocytosis, macropinocytosis and several clathrin-independent pathways [3]. Bacteria exploit the endocytosis process to deliver PFT inside the host cells [2,4]. In response, infected cells have developed several mechanisms to repair the loss of integrity of the membrane caused by the PFT to counteract this strategy. This restoration capability is usually dependent on the rate and duration of the injury.

Endocytosis promotes membrane sealing in response to the PFT, streptolysin O, and perforin in a Ca^2+^-dependent and dynamin-independent mechanism in kidney and HeLa cells [5]. HaCat and Cos7 cells induce endocytosis and exocytosis to survive an α-toxin in a Ca^2+^-independent and dynamin-dependent mechanism [4]. A wounded membrane repair response has also been reported to seal the pore, provoked by perforin. In this process endosomes and lysosomes donate membranes in a Ca^2+^-dependent manner [6].

Related to Bt toxins detoxification, Griffitts and co-workers [7] reported that Cry5B toxin triggers an endocytic mechanism via specific receptors. This study used *Caenorhabditis elegans* and rhodamine-labeled Cry5B toxin to demonstrate, by fluorescence microscopy, that the toxin binds to the nematode gut cells via receptors before being endocytosed [7]. Supporting that previous observation, Los *et al.* [8] reported that increased levels of endocytosis mediated by Rab5 and Rab11 are required to restore plasma membrane integrity in *C. elegans* gut epithelium in response to Cry5B. To date, there are no reports demonstrating that Cry toxins are endocytosed in insect cells or whether the endocytic pathway has a role in detoxification.

Bacteria protein toxins affect the actin cytoskeleton using different strategies. A group of toxins, such as the binary and large clostridial glucosylating toxin, and the Tc toxins of *Photorhabdus luminescens* directly target the actin molecule [9]. Another group interacts with actin-binding proteins to regulate actin cytoskeleton function during internalization [10].

Pore forming toxins can interact directly with actin to enhance actin polymerization *in vitro* [11] or indirectly to promote toxin oligomerization and endocytosis [12]. Interestingly, it has been identified that actin can bind to Cry, in Lepidopteran and Dipteran larvae [13,14]. Based on proteomics studies, it has been reported that Cry toxins affect actin accumulation in *A. aegypti* and *Helicoverpa armigera* [14,15]. The *Aedes aegypti* proteomic profile study showed that actin protein family members are differentially up- or down-regulated in response to Cry11Aa intoxication. One of these actin genes (Accession Number: AAEL005961) was upregulated two times after treatment with sub-lethal doses of Cry11Aa toxin in larvae. Based on those results, it has been suggested that actin may have a role in the toxin mode of action [16].

Here, we characterized the endocytic mechanism triggered by sub-lethal doses of Cry11Aa and Cry1Ab toxins that are active against Diptera and Lepidoptera, respectively, in an *A. aegypti* Mos20 cell line.

Our results showed that Mos20 cells internalized both toxins independently of their specificity. This finding suggests that endocytosis is a general mechanism that insect cells use to cope with pore forming toxins independently of their toxicity. This general endocytic mechanism is mediated by clathrin and flotillin. Our results also demonstrated that low doses of toxin trigger early and recycling endocytosis, similar to the response reported for higher doses of PFT-dependent remodeling of the membrane [8,17]. Here, we also showed that Cry toxins are not degraded in lysosomes. Remarkably, we found that only Cry11Aa toxin, which is toxic to mosquitoes, interacts with actin. Moreover, when the actin gene is silenced, Mos20 cells become hypersensitive to the Cry11Aa toxin, suggesting that actin is an important participant in a specific defense mechanism. Understanding the defense mechanisms employed by the cells in response to Bt Cry toxins can provide tools to design better bio-insecticides to control disease vectors.

## 2. Results and Discussion

### 2.1. Both Cry11Aa and Cry1Ab Toxins Are Internalized into Mos20 Cells at Sub-Lethal Doses

Mos20 cells were exposed to Bt toxins at low doses with the intention to maintain cellular integrity and function and to analyze the role of different endocytosis-related proteins during the intoxication process.

**Figure 1:**
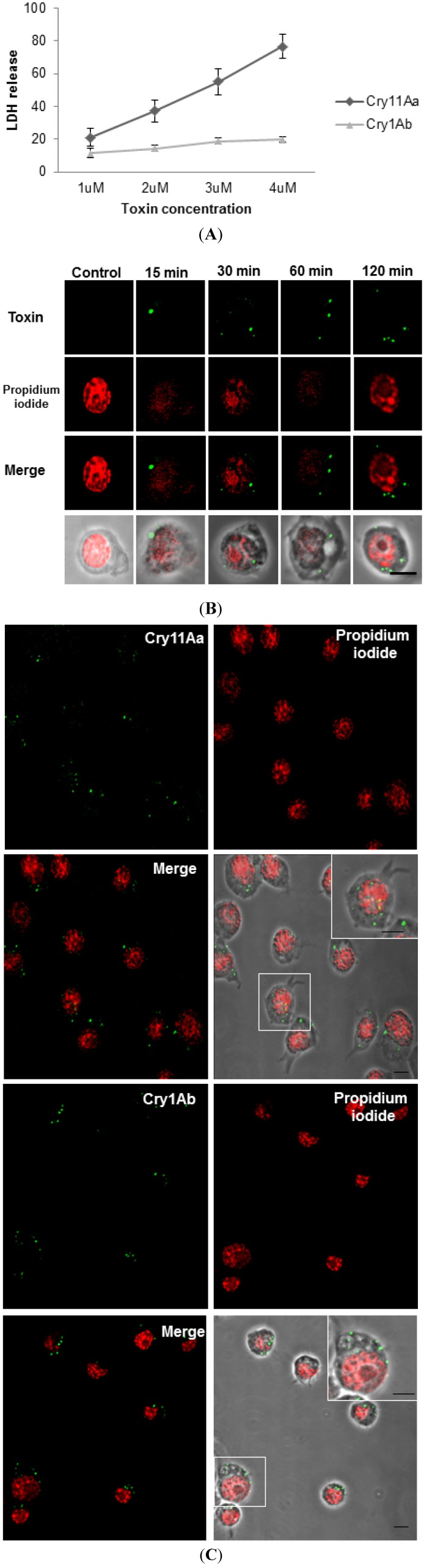
(**A**) Mos20 cells are susceptible to the Cry11Aa toxin. The liberation of lactate dehydrogenase (LDH) into the medium was measured after incubating of Mos20 cells for 6 h with toxins. For comparison, Mos20 cells were exposed to several doses of Cry1Ab. The percentage of liberation was determined by the activity in the sample divided by the activity from cells lysed with Triton X-100. The values shown are the mean of biological replicates (n = 3) and ± SEM; (**B**) Immunolocalization of Bt Cry toxins in the Mos20 cell line in a time-dependent manner. The internalization of Cry11Aa through the cell was observed at 15, 30, 60 and 120 min. Alexa Fluor 488-conjugated secondary antibodies (green) were used for fluorescence confocal microscopy; (**C**) Cry toxins are localized inside Mos20 cells. Mos20 cells were treated for 60 min with 0.1 μM of Cry toxin. Nuclei were stained red with propidium iodide (594 nm).

First, we tested the susceptibility of Mos20 cells to different concentrations of Cry11Aa and Cry1Ab toxins using a viability assay (lactate dehydrogenase (LDH)). The lethal concentration 50 (LC_50_) of Mos20 cells to the lytic mosquito-specific Cry11Aa was 2.8 μM. In contrast, the Lepidopteran-specific toxin, Cry1Ab, was non-lytic at all of the concentrations tested, as expected (Figure 1A). We therefore established 0.1 μM of toxin (LC_2_) to study Cry11Aa endocytosis in this cellular model and whether mosquito cells were able to contend with Cry toxins as proposed for *C. elegans* [8]. An equivalent amount of Cry1Ab (0.1 μM) was used for comparison.

To determine how the Cry11Aa toxin, at low doses, was internalized, Mos20 cells were treated with 0.1 μM of Cry11Aa, and its localization was followed over several minutes with a specific Cry11Aa polyclonal antibody and with an Alexa 488 fluorescent secondary antibody (Figure 1B,C). Cells were imaged by confocal microscopy, and a central slice from a z stack was reported.

During the initial 15 min of treatment, Cry11Aa toxin accumulated outside the Mos20 cellular membrane. At longer times, Cry11Aa started to accumulate inside the cells as small aggregates that grew larger over time. Two hours later, the integrity of the cellular membrane was only disrupted in 2% of the cell population, and the Cry11Aa aggregates were more clearly detectable inside the cells. Nuclei were stained with propidium iodide as the reference for cell size and cell integrity (Figure 1B).

The localization of Cry1Ab toxin was also analyzed using a specific Cry1Ab polyclonal antibody and Alexa 488 fluorescent secondary antibody (Figure 1C). Unexpectedly, after one hour, Cry1Ab formed aggregates that were also observed inside the cell in a similar way to Cry11Aa, suggesting that at low doses, both the specific and non-specific toxins, Cry11Aa and Cry1Ab, were internalized independently of their toxicity to the cell line (Figure 1C).

### 2.2. Clathrin-Dependent and Clathrin-Independent Mechanisms Are Involved in the Internalization of Cry11A

**Figure 2:**
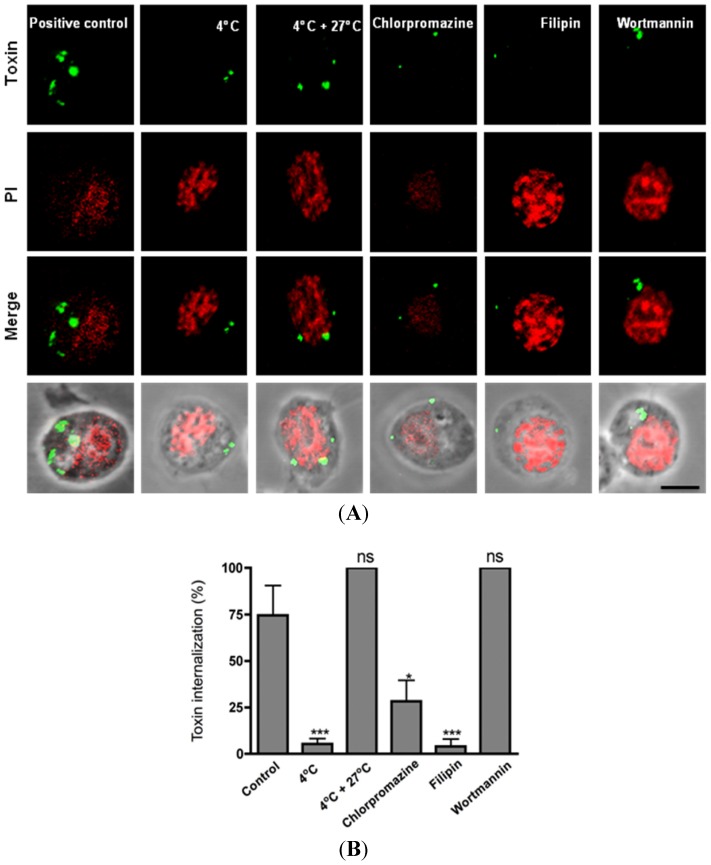
Clathrin-dependent and clathrin-independent mechanisms are involved in the cellular internalization of Cry11Aa. (**A**) For immunofluorescence assays, Mos20 cells were pre-incubated at 27 °C in the presence of endocytic inhibitors. After 30 min, Cry11Aa toxin was added to the cells in the continuous presence of inhibitors, and the cells were incubated for 1 h at 27 °C. Cells were then fixed, permeabilized and detected with a Cry11Aa primary antibody and stained with an Alexa 488-labelled secondary antibody (green). Nuclei were stained red with propidium iodide (594 nm); (**B**) Histograms represent the average proportion of internalization of Cry11Aa-Alexa 488 fluorescence within each cell. Internalization, visualized as dots, was recorded and calculated by the software, ImageJ [22]. The values were normalized to the percent of total internalization in cells without the inhibitor. Data for each condition are presented as the mean of three independent experiments. Statistical significance between chemical and physical treatments and the control were calculated according to the Student’s *t* test (ns, no significance; * *p* < 0.05; *** *p* < 0.05).

Our knowledge regarding the cellular response when insect cell lines are exposed to sub-lethal doses of Cry toxins is very limited. In our model, at low doses, the specific Cry11Aa or the non-specific Cry1Ab toxins were capable of penetrating the cells, but did not lead to cell death 1 h after treatment. To find distinctive characteristics of the cell response to intoxication, we analyzed whether Cry11Aa toxin entered the cell using an endocytic pathway. Mos20 cells were incubated for 1 h in the presence of Cry11Aa at 4 °C, a temperature at which the membrane traffic is reduced [18]. The internalization of Cry11Aa diminished 80% compared to the control at 27 °C. Internalization of Cry11Aa toxin was recovered 100% when the cells were returned to 27 °C (Figure 2A,B). This result suggests that the toxin requires an optimal cell metabolism for internalization, and endocytosis may be involved in this process.

To evaluate which endocytic pathway might be involved in this process, different commonly used endocytic inhibitors were tested, and Cry11Aa internalization was measured as described above. The Mos20 cell line was treated with chlorpromazine (10 μg/mL), a cationic amphiphilic drug that is used to inhibit clathrin-coated pit formation [19], to determine whether Cry11Aa toxin used the clathrin-dependent endocytic pathway to enter the cell. Pre-treatment of Mos20 cells to chlorpromazine for 30 min decreased the internalization of Cry11Aa 70% compared with the control cells, which were defined as 100% internalization (Figure 2A,B). These observations support a role for clathrin in Cry11Aa endocytosis. Figure 2A shows representative images of the toxin aggregates.

To investigate whether another endocytic pathway may be involved in the internalization of Cry11Aa, we tested the effect of filipin, a chemical inhibitor of several cholesterol-related endocytic pathways [20]. Pre-treatment of Mos20 cells to filipin (5 μg/mL) for 30 min diminished the internalization of Cry11Aa to approximately 80% when compared with the control cells (Figure 2B). These observations suggested that cholesterol-dependent endocytosis is also involved in the internalization of Cry toxins.

Cholesterol-dependent endocytosis is usually related to caveolin. The ortholog to the human *caveolin* gene is not present in the *A. aegypti* genome; however, it has been reported that flotillin is enriched when caveolin is absent from lipid rafts. Based on their high identity to caveolin1, it has been suggested that flotillin plays a role in the ordering of lipids in an analogous manner to caveolin [21].

Finally, a pretreatment of Mos20 cells with wortmannin (1 µM), an inhibitor of phosphoinositide 3-kinase that impairs macropinocytosis [23,24], did not affect Cry11Aa internalization, suggesting that macropinocytosis does not contribute to Cry11Aa internalization (Figure 2A,B).

In conclusion, treatment with drugs, such as chlorpromazine and filipin, that affect endocytic pathways prevents Cry11Aa delivery into the cells. These observations suggest that both clathrin-dependent and clathrin-independent endocytic mechanisms participate in Cry11Aa entry into Mos20 cells. Clathrin-dependent endocytosis can be continuously activated in different cell lines, but the clathrin-independent pathways require a signaling process for activation, which can be triggered by cargo recognition [3]. Based on our observations, Mos20 cells can use more than one endocytic pathway to respond appropriately to external stimuli. Supporting our results, Bayyareddy *et al.* reported that flotillin might interact directly with Cry4Ba toxin (another Bt toxin with toxic activity against mosquitoes), as assessed by mass spectrometry and ligand blot experiments [14,25]. Recently, it was reported that Cry4Ab was associated with lipid rafts enriched in flotillin and the aminopeptidase receptor [25]. Our observation provides support to the proposed model in which lipid rafts and their associated proteins have a role during the Cry intoxication process.

### 2.3. Cry Toxins Colocalize with Clathrin and Flotillin in the Mos20 Cell Line

**Supplementary Figure 1:**
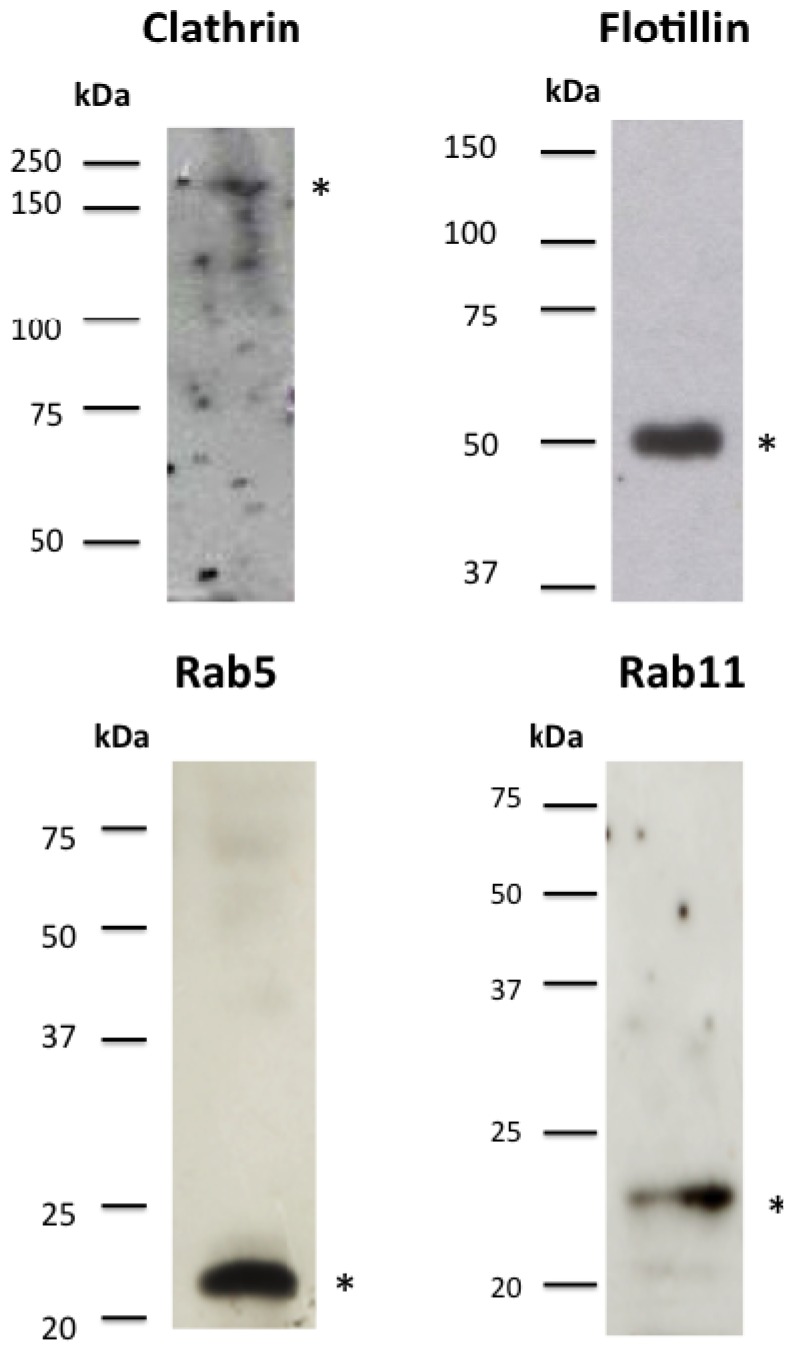
Clathrin, flotillin, Rab5 and Rab11 human antibodies were able to detect homologous proteins from *A. aegypti* Mos20 cells in Western blot experiments. The total protein extract of Mos20 cells was obtained by sonication in lysis buffer, and 40 μg of protein were separated in a 12% SDS-PAGE gel and transferred onto a nitrocellulose membrane. Immunodetection was performed as described in Materials and Methods. * Denotes specific proteins.

To further explore whether Cry toxins enter the cell using a different endocytic pathway, we studied the colocalization of clathrin or flotillin raft-associated proteins with Cry11Aa and Cry1Ab by confocal microscopy using commercial antibodies. We took advantage of genomic information to compare cytoskeletal and endocytic proteins between *A. aegypti* and humans. Given the high levels of identity between human and *A. aegypti* genes (clathrin, 81%; flotillin, 66%), we used human antibodies directed to clathrin and flotillin. Results showed that clathrin and flotillin proteins were detected specifically in *A. aegypti* Mos20 cells byWestern blot (WB) experiments (Supplementary Figure 1).

**Figure 3:**
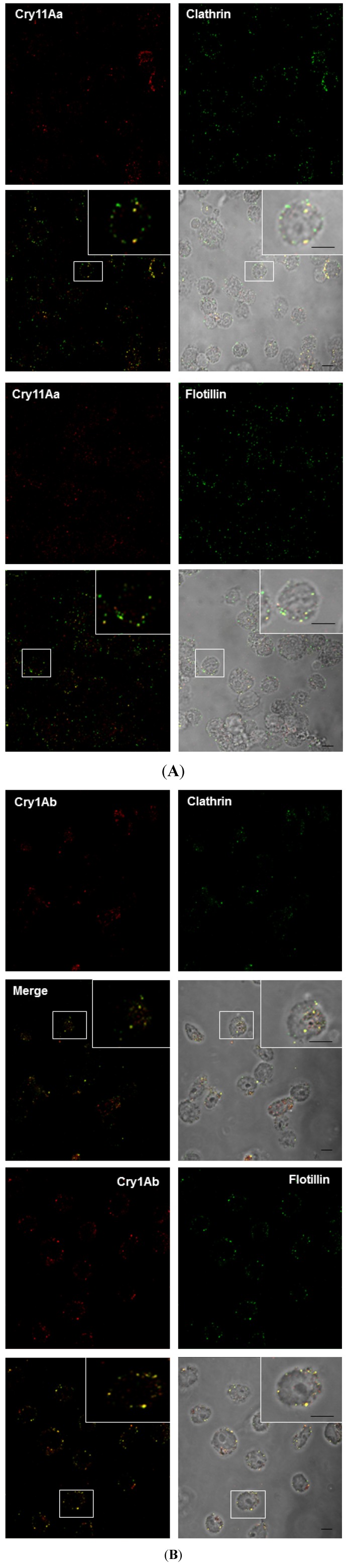
The clathrin- and flotillin-mediated endocytotic pathways are the predominant pathways for Cry toxin internalization in Mos20 cells. Mos20 cells were treated for 1 h with 0.1 μM of Cry11Aa or Cry1Ab toxins. The cells were then fixed, permeabilized, treated with a specific primary antibody and stained with Alexa 594 secondary antibody (red) for the Cry toxin and Alexa 488 secondary antibody (green) for clathrin or flotillin. Immunocolocalization was observed between clathrin (above) and flotillin (below) and (**A**) Cry11Aa toxin or (**B**) Cry1Ab toxin. All images were obtained using a Zeiss confocal microscope S M510 Meta, using a Plan Neofluar 100X/1.3 Ph3 objective. Scale bars: 5 μm.

The immunolocalization of clathrin, flotillin and Cry toxins was determined in Mos20 cells after 30 min of incubation with Cry11Aa or Cry1Ab. Clathrin and flotillin were visualized using specific primary antibodies that were visualized with Alexa 488 or Alexa 594 secondary antibodies, respectively. We observed that both clathrin and flotillin colocalized with Cry11Aa and Cry1Ab in the cytoplasm to a similar extent (Figure 3A,B).

The colocalization of Cry11Aa or Cry1Ab with clathrin and flotillin supports the hypothesis these toxins enter Mos20 cells via clathrin- and cholesterol-dependent pathways and reinforce the idea that Cry toxin internalization is not related to toxicity, because both toxins crossed the Mos20 membrane surface independent of their specificity to the target cells.

### 2.4. Rab5 Colocalizes with Cry11Aa and Cry1Ab

The endocytic pathway is characterized by the participation of several vesicular organelles, including early/sorting endosomes, recycling endosomes, late endosomes and lysosomes. The endosomal system may uptake extracellular molecules and ligands that can be recycled to the Golgi apparatus, the trans-Golgi network and the plasma membrane. Molecules that are not recycled are degraded in the lysosomal system [26,27].

**Figure 4:**
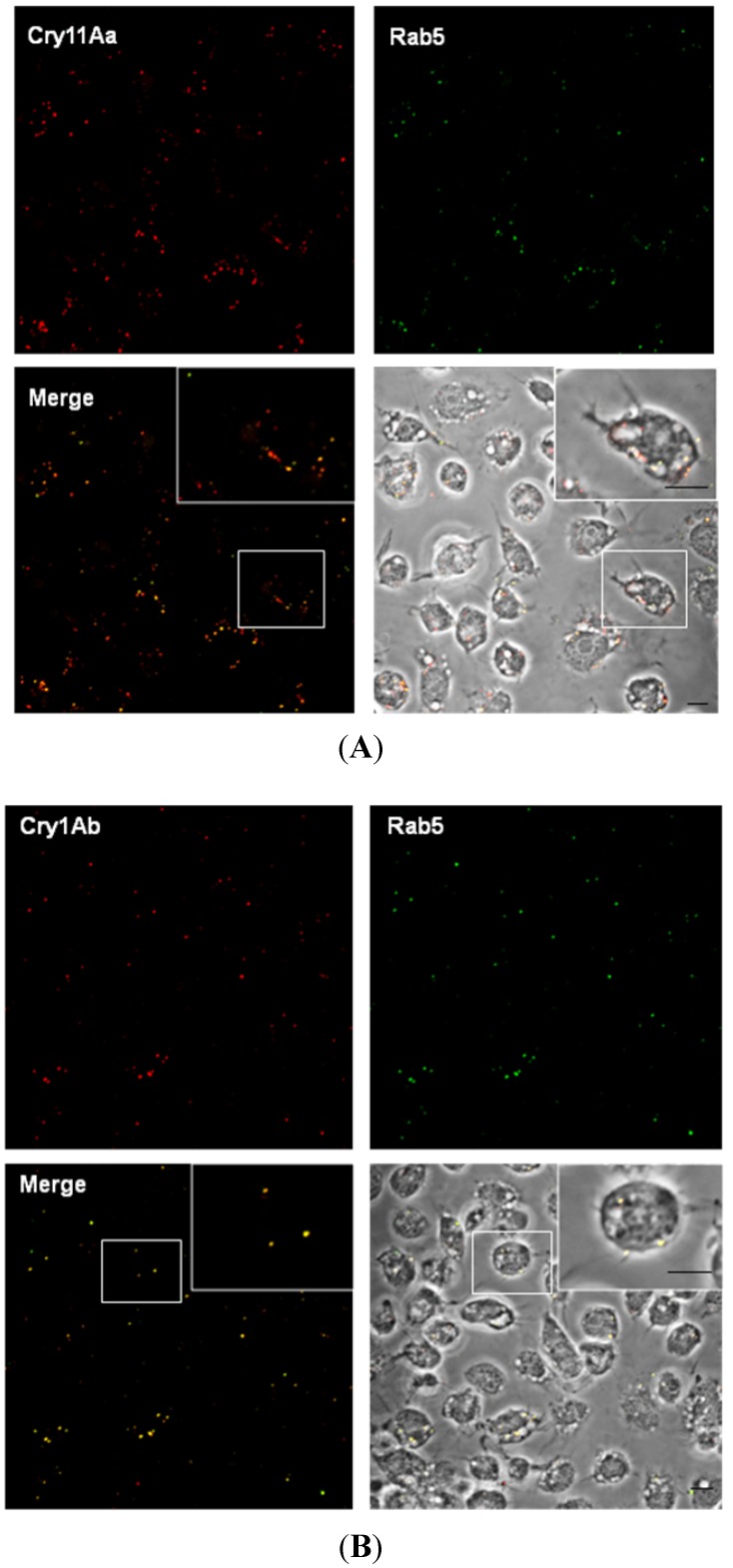
Rab5 colocalizes with Cry11Aa and Cry1Ab. Mos20 cells were treated for 30 min with 0.1 μM of Cry toxins. Cells were then fixed, permeabilized, treated with primary antibody and stained with Alexa 594 secondary antibody (red) for Cry toxin and Alexa 488 secondary antibody (green) for Rab5. Cells were imaged via confocal microscopy, and a central slice from a z stack is shown. (**A**) The left panel shows merged images: Rab5 (green) and Cry11Aa (red). The right panel shows the merge of yellow images on a bright field; (**B**) Colocalization of Rab5 (green) and Cry1Ab (red). Panels are shown similarly as described above. Scale bars: 5 μm.

The Rab family of small GTPases is involved in most endocytic events and regulates cargo traveling through the cell. Rab5 is localized in early endosomes and regulates the fusion between endocytic vesicles and the early endosomes [28]. To determine whether Rab5 is localized with Cry toxins into the cell, we followed Rab5 using human antibodies (77% identity with *A. aegypti* Rab5) in total protein extracts from control (untreated) or Cry11Aa-treated Mos20 cells by WB. We found that Rab5 is an abundant protein in Mos20 cells (Supplementary Figure 1). The colocalization of Rab5 with Cry toxins was determined in Mos20 cells after 30 min of incubation with Cry11Aa or Cry1Ab using specific antibodies and fluorescent secondary Alexa 488-labeled antibodies. Rab5 colocalized with Cry11Aa or Cry1Ab in the cell cytoplasm near the cell membrane (Figure 4A,B). Therefore, Cry11Aa and Cry1Ab toxins may use Rab5 as a common mechanism for transportation into early endosomes. In Mos20 cells, the endocytosis at low doses of Cry toxins may be related to a basal immune response and not to their toxicity. Our observations differ from previously reported data in which the defense response to high doses of Cry5-specific toxins is Rab5 endocytosis-dependent in *C. elegans* [8].

### 2.5. Rab11 Colocalizes with Cry11Aa and Cry1Ab

**Figure 5:**
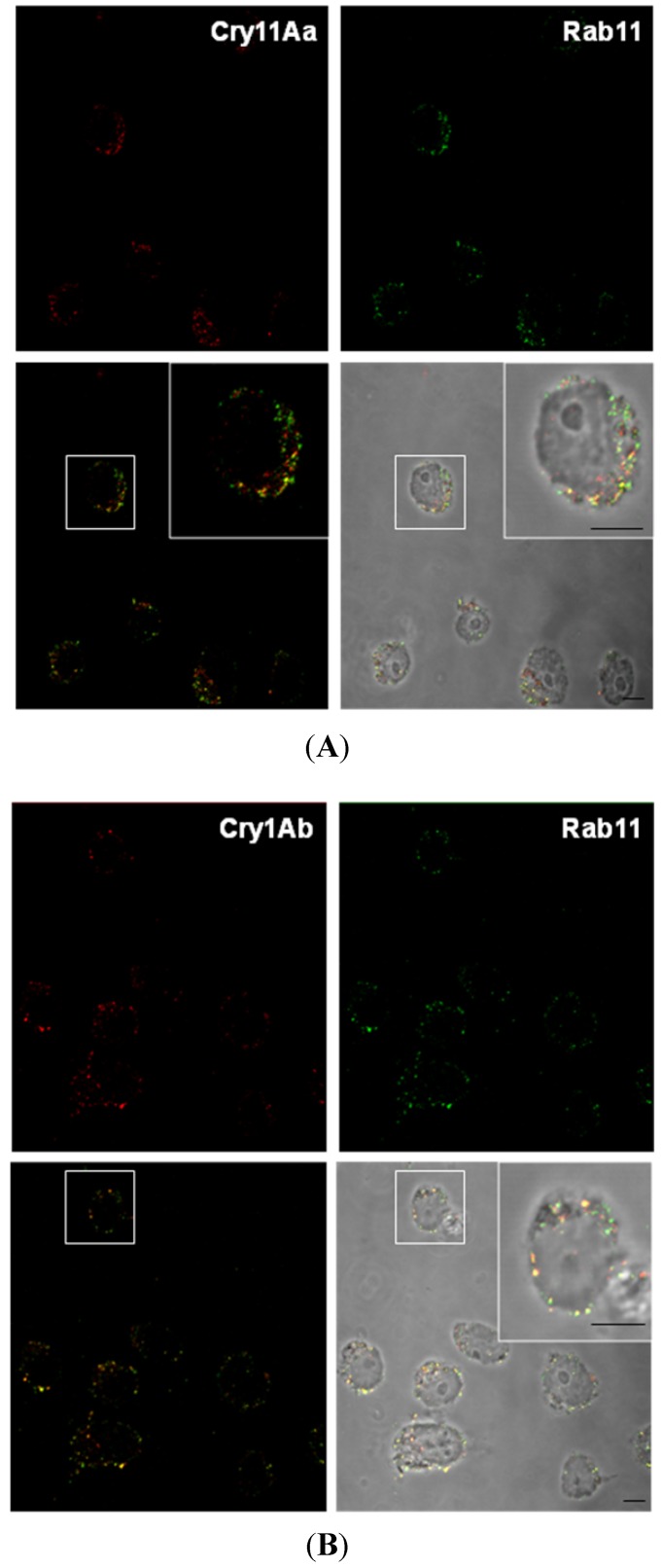
After internalization, Cry toxins reach recycling endosomes. Mos20 cells were treated with Cry toxins for 90 min. The cells were processed for confocal microscopy analysis using a primary antibody to toxins and a marker of slow recycling endosomes. The primary antibodies were visualized with Alexa 594 secondary antibody (red) for Cry toxin and Alexa 488 secondary antibody (green) for Rab11. (**A**) Cry11Aa fluorescent signal colocalized with Rab11, a typical marker of recycling compartments; (**B**) colocalization was detected between the Cry1Ab toxin and Rab11. Scale bars: 5 μm.

A large amount of sorting and recycling occurs in the early/sorting endosomes. This pathway can rapidly or slowly return proteins to the plasma membrane via recycling endosomes [29]. Rab11, a Rab-GTPase, guides vesicles from the recycling endosome to the plasma membrane [30]. To determine whether Rab11 was involved in Cry11Aa trafficking, we evaluated Rab11 in total protein extracts from Mos20 cells by WB, using specific human antibodies (83% identity with *A. aegypti*; Supplementary Figure 1). Then, the colocalization of Rab11 with Cry toxins was determined in Mos20 cells after 90 min of incubation with Cry11Aa or Cry1Ab using human antibodies. Rab11 colocalized with Cry11Aa (Figure 5A) or Cry1Ab (Figure 5B) in the cytoplasm near the cell membrane. Mos20 cells may use the Rab5 and the Rab11 pathway as a common mechanism for the early endocytosis and the expulsion of Cry11Aa and Cry1Ab toxin from recycled endosomes to the surface membrane. However, only a fraction of Cry toxins colocalized with Rab5 or Rab11: Cry11Aa-Rab5, 27%; Cry11Aa-Rab11, 13%; Cry1Ab-Rab5, 50%; Cry1Ab-Rab11, 25%. Then, it is possible that Cry toxins interact with another Rab transporters. Moreover, RNAi silencing of Rab5 and Rab11 experiments will support its specific role in Cry toxin recycling.

Intracellular organelles reseal the plasma membrane by membrane exocytosis, and the secreted vesicles replace the damaged area and repair membrane integrity after attack by a toxin, [31]. A recent work [8] showed that Cry5B toxin from Bt can activate endocytosis in *C. elegans* intestinal cells, and this process is under the regulation of Rab5 and Rab11 GTPases, suggesting the involvement of the recycling pathway as a defense mechanism against PFT intoxication. In the case of *C. elegans* intestinal cells, a defense mechanism is triggered. In the case of the Mos20 cell model, the endocytic process that we have observed may be not related to a defense response, given that it is not specific towards specific or non-specific Cry proteins. A possibility is that this response is a non-specific endocytosis mechanism used by the cell to remove toxins that contact the cellular membrane.

### 2.6. Cry11Aa and Cry1Ab Do Not Colocalize with Lysosomes

Toxins with activity against mammalian cells induce defense mechanisms, which include the remodeling of the plasma membrane and changes to the intracellular traffic pathways. For example, the toxin, SLO (streptolysin-O *Streptococcus* spp.), induces its own endocytosis to be transported to degradation pathways in HeLa, HEK293 and NRK cells [5].

**Figure 6:**
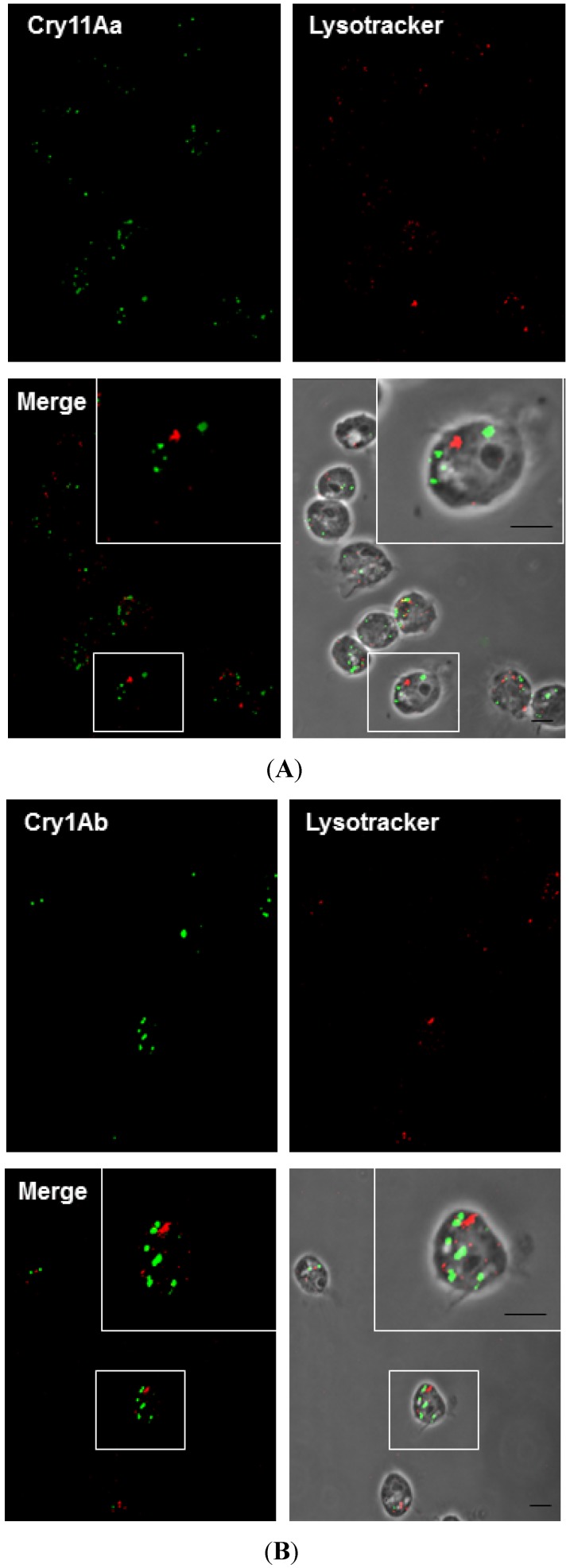
Cry11Aa and Cry1Ab do not colocalize with lysosomes. Mos20 cells were treated with Cry toxins for 2 h. The cells were processed for confocal microscopy analysis using primary antibody against toxins labeled with Alexa 488 secondary antibody (green), and LysoTracker (red) (1 μg/mL) was added to the medium 5 min before the end of the treatment. No colocalization was found with Cry11Aa (**A**) or Cry1Ab (**B**) and LysoTracker (late endocytotic/lysosomal compartments marker). Scale bars: 5 μm.

Taking into account that the common intracellular destination for endocytosed material is the lysosome, we looked for colocalization of Cry11A or Cry1Ab within lysosomes using the acid-tropic fluorescent dye, LysoTracker, which selectively stains lysosomes in living cells. No colocalization of toxin treatment was observed after 2 h, indicating that the toxin was not directed to this degradation pathway (Figure 6A,B). This finding is notable, because it has been reported that most cargo is degraded within the cell [32,33]. These results suggest that toxins are associated with recycling endosomes, but not with lysosomes. Therefore, endocytosed Cry11Aa and Cry1Ab may be subjected to other degradation or exocytosis pathways.

### 2.7. Actin Is Required for the Toxicity of Cry11Aa

**Figure 7:**
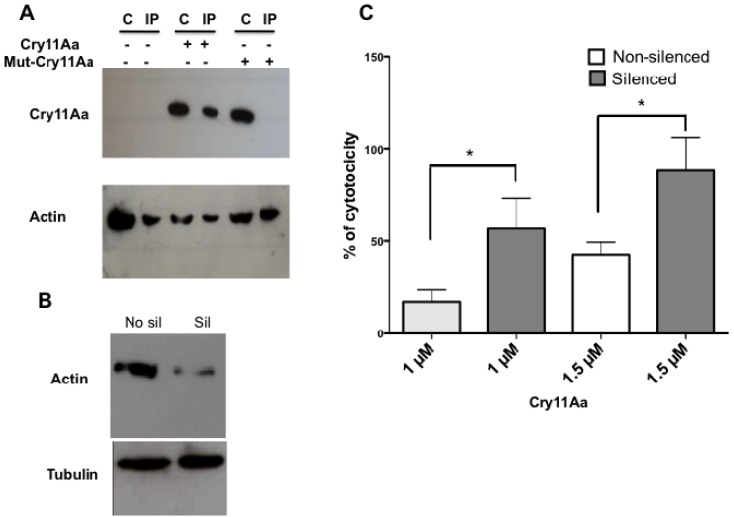
Toxicity of Cry11Aa is actin dependent. (**A**) Immunoprecipitation of endogenous actin from Mos20 cells. Experiments were performed with 1 mg of Mos20 cell protein previously treated with a lethal concentration (LC_2_) of Cry11Aa. Samples were immunoprecipitated with actin antibody coupled-Sepharose, separated by SDS-PAGE, and immunoblotted with anti-Cry11Aa or anti-actin antibodies. A protein sample (50 µg) was co-migrated for comparison. (**B**) Inhibition of actin expression by RNA interference. To silence the expression of actin, Mos-20 cells were transfected with 1 µg of actin dsRNA with Effectene. Assays were performed two days after transfection. Using Western blot, the efficiency of actin silencing was assessed in cell protein extracts. (**C**) After actin knockdown, the cells were treated with Cry11Aa toxin, and cell viability was evaluated quantifying the release of lactate dehydrogenase (LDH). Values are presented as the mean and +/− SEM. of biological replicates (n = 3). Statistical significance between silenced and non-silenced cells was calculated according to the Student’s *t* test (* *p* < 0.05).

Actin is indispensable during several stages of endocytosis, including invagination, neck elongation of the endocytic vesicle, fission and movement away from the plasma membrane [34,35]. Interestingly, it has been reported that the actin cytoskeleton reorganized one of the two anthrax toxin receptors at the cell surface to help the endocytosis of the anthrax toxin [12]. Looking for a mechanism involved in Cry11Aa lytic activity in Mos20 cells, we evaluated whether Mos20 cell endogenous actin interacted with Cry11Aa, a non-toxic, V142D-Cry11Aa, by co-immunoprecipitation assays. Immune complexes between endogenous actin and internalized toxins were analyzed. Immunoprecipitation of endogenous actin led to the co-immunoprecipitation of internalized Cry11Aa using antibodies against Cry11Aa by WB assay. To analyze if the co-immunoprecipitate actin-Cry11Aa complex depends on Cry11Aa toxicity, a non-toxic V142D-Cry11Aa mutant, previously reported [36], that is inactive against *A. aegypti* larvae and Mos 20 cells (data not shown), was internalized on Mos20 cells, and the immunocomplex was analyzed in WB using Cry11Aa antibodies. Our co-immunoprecipitation assays demonstrate that the wild-type Cry11Aa toxin and actin interact in the same molecular complex. This interaction is no longer detected, however, when an inactive Cry11Aa mutant protein was used (Figure 7A). Based on these data, we suggest that actin and Cry11Aa participate in the same complex during the initial steps of toxic interaction with the cell membrane. This interaction is specific and depends on toxicity. Interestingly, related toxins, Cry4Ba and Cry1Ac, can also interact with actin in *A. aegypti* and Lepidoptera, respectively, [13,14,34,35] suggesting that actin is a common component of cell response to Cry toxins.

The interaction between Cry11Aa toxin and actin would occur after toxin penetration, on the cytoplasmic side of the membrane. For Cry toxins, an umbrella model of membrane toxin insertion was proposed, where only the hairpin conformed by helices α-4 and α-5 is inserted into the membrane [37,38,39,40]. Cry11Aa may interact directly with actin or other actin-binding proteins through the loop between α-4 and α-5 that remains in the cytoplasmic side of the cell membrane. Another possibility is that actin binds to a receptor, like cadherin or aminopeptidase, which has an intracellular domain [41].

#### Mos 20 Cells Are More Susceptible to Cry11Aa If Actin Was Silenced

To evaluate whether actin was involved in the Cry11Aa toxicity, first, we silenced the *actin* of Mos20 cells by RNAi. WB and densitometry were used to evaluate the reduction of the actin expression. The result was that actin was 70% silenced (Figure 7B). Next, we tested the susceptibility of actin-silenced Mos20 cells treated with one or 1.5 μM of Cry11Aa toxin using a viability assay (LDH) (Figure 7C). *Actin*-silenced Mos20 cells became more sensitive to the toxic action of Cry11A, since lower doses than an LC_50_ were sufficient to kill more than 50% of silenced cells. This result supports the specificity and the dependences of actin for Cry11Aa toxicity in Mos20 cells and is consistent with a previous report that showed that the *actin*-silenced larvae became more sensitive to the insecticidal action of Cry11A toxin [16]. Based on our results, we suggest that actin has a defense role against Cry11Aa in insect cells.

## 3. Experimental Section

### 3.1. Cell Lines

The *A. aegypti* Mos20 cell line was generated from completely macerated *A. aegypti* larvae [17]. The Mos20 cell line was kindly provided by Dr. Humberto Lanz (Centro de Investigaciones Sobre Enfermedades Infecciosas, I.N.S.P., City, México). Cells were maintained in MEM, supplemented with a commercial mix of vitamins (100 mg/L choline chloride, 100 mg/L D-calcium pantothenate, 100 mg/L folic acid, 100 mg/L nicotinamide, 100 mg/L pyridoxal hydrochloride, 10 mg/L riboflavin, 100 mg/L thiamine hydrochloride and 8500 mg/L sodium chloride) (Invitrogen, Carlsbad, CA, USA) and 10% fetal bovine serum (Gibco, BRL, Grand Island, NY, USA). Cultures were maintained at 27 °C with atmospheric CO_2_ concentration (not controlled), and cells were passaged at a 1:3 dilution every 3 to 4 days.

### 3.2. Toxin Purification and Processing

*B. thuringiensis* strains harboring either pHT315-Cry1Ab or pCG6-Cry11Aa [42,43] plasmids were grown at 30 °C in HCT medium with 10 mg/mL erythromycin until complete sporulation. Cry protoxin crystals were observed with phase contrast microscopy and were purified by sucrose gradients [44]. Protoxin Cry11Aa was solubilized in 100 mM NaOH and 0.2% β-mercaptoethanol for 8 h at 4 °C, and protoxin Cry1Ab was solubilized in an alkaline buffer of 50 mM Na_2_CO_3_ and 0.2% β-mercaptoethanol, pH 10.5. Both toxins were activated with trypsin at a mass ratio of 1:20 (w/w trypsin/protoxin) for 4 h at 37 °C. The pH was stabilized to pH 8.5 with equal volumes of 1 M Tris HCl, pH 8. Finally, the trypsin was inactivated with 1 mM phenylmethylsulfonyl fluoride (final concentration).

### 3.3. Cell Viability Measurement

Cell viability was evaluated by quantifying the amount of lactate dehydrogenase (LDH) released with the Citotox96^®^ non-radioactive cytotoxicity assay (Promega, Fitchburg, WI, USA), following the manufacturer’s instructions. Briefly, 100 mL of Mos20 cell suspension, containing approximately 100,000 to 140,000 cells, was incubated 1 h at 27 °C in a 96-well plate. Activated toxin was added to a final concentration of 1–4 μM, and the plate was incubated 6 h at 27 °C. The samples were read at 490 nm in an ELISA spectrophotometer to determine the concentration of formazan released into the medium. This concentration is related to the amount of LDH released by cells.

### 3.4. Inhibition Treatments

A confluent cell culture that was grown on a coverslip was placed in one well of a 6-well plate. Cells were incubated with 1 mL of medium containing chlorpromazine (10 μg/mL), filipin (5 μg/mL) or wortmannin (1 μM), and the cells were incubated for 30 min at 27 °C. After 30 min, a lethal concentration of 2% of Cry toxins (LC_2_ = 0.1 μM) was added, and the samples were incubated for 1 h at 27 °C. To inhibit metabolic activity, samples were incubated for 1 h at 4 °C. A viability control was introduced by incubating one of the metabolically inhibited samples for an additional hour at 27 °C to verify that metabolic activity was restored.

### 3.5. Antibodies

Proteins in Western blot (WB) assays or immunofluorescence (IF) assays were detected using primary antibodies. The following antibodies were used: polyclonal anti-Cry11Aa (dilution 1:25,000); polyclonal anti-Cry1Ab (dilution 1:30,000); polyclonal anti-clathrin (Santa Cruz sc-6640; Santa Cruz, CA, USA; dilution 1:100); polyclonal anti-flotillin (Santa Cruz sc-6579; dilution 1:200); polyclonal anti-Rab5 (Santa Cruz sc-26566; dilution 1:500 WB/1:50 IF); polyclonal anti-Rab11 (Santa Cruz sc-6565; dilution 1:500 WB/1:50 IF); and polyclonal anti-Actin (Santa Cruz sc-1616; dilution 1:500). For secondary indirect detection, we used polyclonal anti-goat or anti-rabbit antibodies coupled with fluorophores Alexa488^®^ or Alexa594^®^ (Invitrogen; dilution 1:1,000) for IF or polyclonal anti-goat or anti-rabbit antibodies coupled to horseradish peroxidase (HRP; Santa Cruz; dilution 1:30,000) for WB analysis.

### 3.6. Immuno Fluorescence Assay

A confluent cell culture grown on a coverslip was placed in a well of a 6-well plate and was incubated for 1 h at 27 °C. Depending on the experiment, the cells were then incubated for an additional 15–120 min with or without Cry toxins at 27 °C. Cells were fixed for 20 min with 4% p-formaldehyde, permeabilized with 0.15% triton X-100 in 1 × PBS containing 1% bovine serum albumin (BSA) for 40 min and blocked for 10 min with 1 × PBS containing 1% BSA. Samples were incubated with primary antibodies overnight (ON) at 4 °C and with a secondary antibody for 1 h at room temperature. Subsequently, some samples were incubated for 5 min with either 1 µg/mL propidium iodide solution (Invitrogen) or 1 µg/mL LysoTracker^®^ solution (Invitrogen) to label either the nuclei or the lysosomes, respectively.

### 3.7. Western Blots Assay

Mos20 cells were sonicated for 1 min at 80% mA in 300 mL of CelLytic M Cell Lysis reagent (Sigma-Aldrich, St. Louis, MO, USA) that was supplemented with 15 mL of Protease Inhibitor Complete (Roche, Mannheim, Germany). Samples were centrifuged for 5 min at 5,000 × *g*. The protein concentration was determined according to the Bradford method with a BSA standard. The supernatant was recovered and stored at −20 °C until analysis. Mos20 cells were homogenized, and 40 μg of protein were solubilized in a sample buffer and resolved on a 12% SDS-PAGE, as described by Laemmli [45]. Proteins were electro-transferred to a nitrocellulose membrane at 350 mA for 90 min. Membranes were blocked ON at 4 °C in 1× PBS, 5% wt/vol non-fat dry milk and 0.1% vol/vol Tween 20. Membranes were then washed 3 times for 5 min in 1× PBS and 0.1% (vol/vol) Tween 20. Incubations with the primary antibody were performed ON at 4 °C, using primary antibodies diluted as described above in 1× PBS and 0.1% vol/vol Tween 20. Membranes were then washed 3 times (5 min each). The primary antibody was detected with HRP-conjugated antibodies, which was followed by enhanced chemiluminescence detection (ECL; GE, Amersham Biosciences, Little Chalfont, Buckinghamshire, UK), as described by the manufacturers. The molecular weight marker used in SDS-PAGE was the Precision Plus Protein Prestained All Blue Standards (Bio-Rad, Hercules, CA, USA). Actin was identified with WB using a similar protocol, and the amount present was used as a loading control.

### 3.8. Inhibition of Protein Expression by RNA Interference

An RT-PCR actin product (AAEL005961) from *A. aegypti* was previously cloned into a pLitmus28i vector as described [46]. To obtain actin dsRNA, a PCR product was obtained with T7 RNA polymerase primer using pLitmus28i-actine construct as a template. PCR product was purified with a QIAquick PCR purification kit (Qiagen, Valencia, CA, USA). *In vitro* transcription of both DNA strands of actin product (190 bp) was performed with T7 RNA polymerase using the HiScribe RNAi Transcription Kit (New England Biolabs), as reported by the manufacturer. To silence the expression of actin, 2.5 × 10^5^ Mos20 cells were transfected with 1 µg of dsRNA previously encapsulated in Effectene transfection reagent (Qiagen, Valencia, CA, USA). Ribosomal protein S3 (*rpS3*) dsRNA was used as a control. Using WB, the efficiency of actin silencing was assessed in cells protein extracts. After actin knockdown, the cells were treated with Cry11Aa toxin (LC_50_), and cell viability was evaluated by quantifying the release of lactate dehydrogenase (LDH) with the Citotox96^®^ non-radioactive cytotoxicity assay (Promega, Fitchburg, WI, USA), following the manufacturer’s instructions.

### 3.9. Co-immunoprecipitation

Immunoprecipitation with the anti-actin antibody was performed using 1 mg of protein of Mos20 cell lysates, previously treated with an LC_2_ of Cry11Aa. The presence of Cry11Aa toxin was revealed with an antibody directed to the toxin; 50 μg of the input was co-migrated for comparison.

### 3.10. Confocal Microscopy

Mos20 cells grown on coverslips were mounted with Citifluor^®^ (Electron Microscopy Sciences, Hatfield, PA, USA) and were analyzed with a Zeiss confocal S M510 Meta microscope, using a Plan Neofluar 100X/1.3 Ph3 oil objective. Alexa Fluor 488 was excited with the 488-nm laser line, and the emitted fluorescence between 500 and 560 nm was measured. Alexa Fluor 594 was excited with the 594-nm laser line, and the emitted fluorescence between 605 and 700 nm was measured. Fluorescence measurements were always performed with the same hardware settings (laser intensity, sampling, acquisition rate, pinhole and photomultiplier settings). To evaluate the internalization of the fluorescent signal, 10 or more cells from at least 2 independent preparations were analyzed for each experimental condition. Internalization, visualized as dots, was recorded and calculated by the software, ImageJ [22]. The percentage of toxin internalization is the ratio of fluorescent dots in the cytoplasm related to the total, represented by dots bound to cytoplasm and membrane.

## 4. Conclusions

After studying the internalization of Cry toxins at low doses in a mosquito cell line, we conclude that Mos20 cells do not discriminate between endocytosis pathways during the internalization of a specific Cry11Aa toxin compared with a non-specific Cry1Ab toxin. Moreover, both toxins are endocytosed by clathrin- and flotillin-dependent mechanisms. The internalized toxins are associated with recycling endosomes, but not with the degradative lysosomes. These observations suggest that other degradative or recycling pathways are involved to counteract toxin invasion independent of its cellular specificity. 

We also conclude that actin has a defense function against Cry11Aa in insect cells. Our data showed an interaction between actin and Cry11Aa, probably through a receptor or another protein. This interaction is related to the toxic process, since it is not observed for non-specific toxins (Cry11Aa mutant and Cry1Ab). Moreover, we propose that the *A. aegypti* Mos20 cell line is a suitable model for the study of endocytic pathways that participate in PFT cell membrane interaction.
